# Supercritical Solvent Impregnation of Different Drugs in Mesoporous Nanostructured ZnO

**DOI:** 10.3390/pharmaceutics11070340

**Published:** 2019-07-15

**Authors:** Mauro Banchero, Sara S. Y. Mohamed, Federica Leone, Francesca Lopez, Silvia Ronchetti, Luigi Manna, Barbara Onida

**Affiliations:** Dipartimento di Scienza Applicata e Tecnologia, Politecnico di Torino, Corso Duca degli Abruzzi, 24, 10129 Torino, Italy

**Keywords:** supercritical carbon dioxide, poorly water-soluble drugs, ibuprofen, clotrimazole, hydrocortisone, zinc oxide, drug adsorption, amorphization

## Abstract

Supercritical solvent impregnation (SSI) is a green unconventional technique for preparing amorphous drug formulations. A mesoporous nanostructured ZnO (mesoNsZnO) carrier with 8-nm pores, spherical-nanoparticle morphology, and an SSA of 75 m^2^/g has been synthesized and, for the first time, subjected to SSI with poorly water-soluble drugs. Ibuprofen (IBU), clotrimazole (CTZ), and hydrocortisone (HC) were selected as highly, moderately, and poorly CO_2_-soluble drugs. Powder X-ray diffraction, Fourier transform infrared spectroscopy, field emission scanning electron microscopy, nitrogen adsorption analysis, and ethanol extraction coupled with ultraviolet spectroscopy were employed to characterize the samples and quantify drug loading. Successful results were obtained with IBU and CTZ while HC loading was negligible, which could be related to different solubilities in CO_2_, drug size, and polarity. Successful SSI resulted in amorphous multilayer confinement of the drug. The mesoNsZnO-IBU system showed double drug loading than the mesoNsZnO-CTZ one, with a maximum uptake of 0.24 g/g. Variation of contact time during SSI of the mesoNsZnO-IBU system showed that drug loading triplicated between 3 and 8 h with an additional 30% increment between 8 h and 24 h. SSI did not affect the mesoNsZnO structure, and the presence of the adsorbed drug reduced the chemisorption of CO_2_ on the carrier surface.

## 1. Introduction

Supercritical fluid technology can be considered one of the most effective alternatives to conventional manufacturing processes of pharmaceuticals especially as far as drug delivery and biomedical applications are concerned [1,2,3]. Its main advantage lies in the fact that organic solvents are replaced with benign fluids such as supercritical CO_2_ (scCO_2_), which is non-toxic, economic, non-flammable, and recyclable. Carbon dioxide creates an oxygen-free protective atmosphere during the manufacturing process and, eventually, a simple depressurization step allows a ready-to-use and organic solvent-free pharmaceutical product to be obtained [4]. Furthermore, the physical properties of this fluid (i.e., viscosity, density, solvent power) can be easily tuned by adjusting the operative temperature and pressure or by adding small amounts of cosolvents [1]. The main drawbacks of this technology are the elevated costs connected with high-pressure operation [2]; however, these could be dampened in the context of a pharmaceutical process where high added-value products are obtained.

Supercritical solvent impregnation (SSI) is among the techniques that can be used to prepare drug delivery vehicles by means of scCO_2_ [1,2]. It consists of dissolving the drug in the supercritical medium, which is brought in contact with the adsorbent material. Different adsorbent materials, such as biocompatible polymers [3,5], cyclodextrins [6,7], and inorganic porous structures [8,9], can be used. When a non-swellable porous matrix is employed, the SSI process is also known as “adsorptive precipitation”, since both adsorption of the drug on the surface of the carrier and drug precipitation upon scCO_2_ depressurization occur [4]. Depending on the drug-drug and drug-matrix interactions, the drug can be confined in the porous structure in its amorphous form [4]. This has gained a great deal of attention in pharmaceutical science, since amorphous drug formulations may result in significant increase of the solubility and dissolution rate of poorly water-soluble drugs [10].

Zinc oxide (ZnO) is a material that is widely used in pharmaceutical topical formulations thanks to its biocompatibility and its intrinsic anti-inflammatory, antimicrobial, and antifungal activity [11,12]. In recent years, ZnO nanostructures have been exploited as anti-cancer, anti-diabetic, or antibiotic drug carriers by many research groups [11,13]. However, the recent review by Gurikov and Smirnova [4] does not include this material among those that can be used to achieve drug amorphization by SSI, and our research group appears to be the only one that has attempted its investigation [14,15]. This may be ascribed to the fact that, even though many ZnO morphologies exhibit high surface areas, they often lack of mesoporosity, which has limited their investigation as drug delivery carriers with respect to other materials, such as mesoporous silica nanoparticles [13]. In fact, it has been reported that mesoporous materials have the ideal pore size (2–50 nm) to be employed as amorphization drug carriers [4].

In this work, a mesoporous nanostructured ZnO (mesoNsZnO) carrier has been synthesized and, for the first time, tested to achieve SSI of poorly water-soluble drugs. With respect to our previous works [14,15], the ZnO here investigated displays a mesoporous structure and conjugates both a high specific surface area and a morphology made of spherical nanoparticle aggregates, which may be considered more suitable for biological applications because it does not involve the toxicological issues of rod-like morphologies [16]. To assess the use of SSI in the prospect of exploiting this mesoNsZnO material as a drug delivery carrier for topical formulations, experiments with three poorly water-soluble drugs were conducted: Ibuprofen (IBU), clotrimazole (CTZ), and hydrocortisone (HC). Whereas IBU is a commonly used non-steroidal anti-inflammatory drug, HC is a well-known anti-inflammatory corticosteroid, and CTZ is a broad-spectrum antifungal drug. All drugs are widely used for topical administration and have successfully been used in scCO_2_-mediated processes [4,9,17]. According to the literature [4], IBU, CTZ, and HC can be classified as highly, moderately, and poorly soluble in scCO_2_, since their solubility (expressed as mole fraction) is ≥10^−3^, included in the 10^−5^–10^−3^ range, and ≤10^−6^, respectively [18,19,20]. A comparison of the drug loading of the three active ingredients is of great interest since it has been reported that low drug solubility in scCO_2_ does not necessarily involve low loading on the carrier [4]. Samples were characterized by means of powder X-ray diffraction, Fourier transform infrared spectroscopy, field emission scanning electron microscopy, and nitrogen adsorption analysis. Ethanol extraction and ultraviolet spectroscopy were employed to quantify the amount of drug loading after SSI. Since the mesoNsZnO-IBU system resulted in the most promising one among those here reported, it was selected to conduct different tests aimed at investigating the role of the contact time during the supercritical treatment on the final drug loading.

## 2. Materials and Methods

### 2.1. Materials

Carbon dioxide with a purity of 99.998% was supplied by SIAD (Torino, Italy). All reactants for the synthesis and characterization of the nanostructured ZnO were purchased from Sigma-Aldrich and used as received without further purification. IBU, CTZ, and HC with purity ≥98% were also purchased from Sigma-Aldrich (Milano, Italy) and used as received. Table 1 reports, for each drug, the chemical structure, the *n*-octanol water partition coefficient (log*P*), and some structure-based predicted properties.

### 2.2. Synthesis of the MesoNsZnO Carrier

The mesoNsZnO carrier was synthesized according to the precipitation method reported by Mitra and coworkers [23]. A total of 14.75 g of zinc acetate dihydrate and 7.4 g of potassium hydroxide were separately dissolved in 60 mL and 32 mL of methanol, respectively. After mixing the two solutions at ambient temperature under constant stirring, the resulting mixture was refluxed at 60 °C and 350 rpm for 72 h to allow the hydrolysis reaction to be completed and the ZnO precipitate to be formed. The precipitate was separated through three 30-min centrifugation cycles at 4000 rpm, each of them being followed by a pure methanol washing step to remove the excess of potassium hydroxide. Eventually, the white precipitate was dried in oven at 50 °C for 24 h.

### 2.3. Drug Loading through SSI

**Figure 1:**
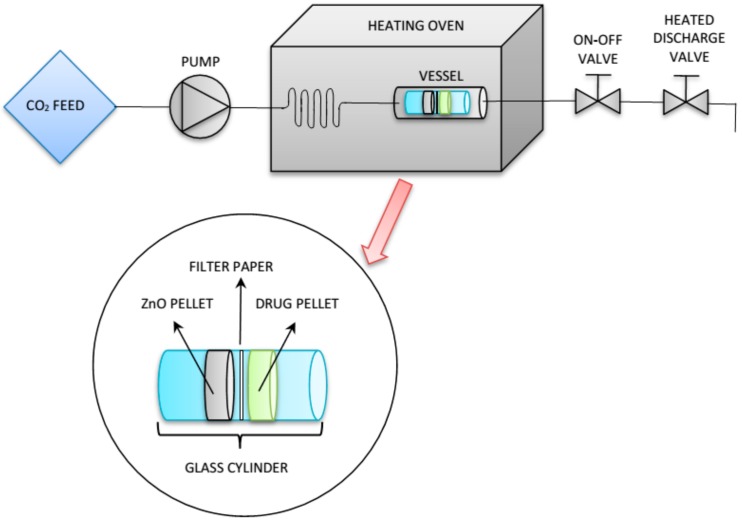
Experimental apparatus for supercritical solvent impregnation (SSI).

SSI of the mesoNsZnO carrier was conducted in a laboratory experimental apparatus (Figure 1). The impregnation procedure consisted in contacting a pellet of the carrier (100 mg) with a pellet of the drug (100 mg) in a static atmosphere of scCO_2_ at constant temperature and pressure [9]. Tests with each drug were conducted in different runs.

The drug and the carrier pellets were introduced in a 1-cm diameter glass cylinder. A disc of filter paper was placed between the two pellets to avoid their contact and allow an efficient recovery of the ZnO sample at the end of the drug loading procedure. The glass cylinder was placed inside a stainless-steel vessel that was filled in with scCO_2_ at the prefixed pressure and kept inside an oven to guarantee the temperature constancy during the entire SSI process. At the end, the on-off valve was opened, and the vessel was depressurized by means of a heated discharge valve. Further details of the experimental apparatus and the impregnation procedure can be found in a previous work [24].

Table 2 reports the working conditions (temperature, pressure, and contact time) of the SSI for the different mesoNsZnO drug systems. As it has already been mentioned in the introduction section, IBU, CTZ, and HC can be classified as highly, moderately and poorly soluble in scCO_2_, which corresponds to different orders of magnitude of the solubility value [4] and of the corresponding temperature and pressure ranges [18,19,20]. This requires that different values of the working temperature and pressure are adopted for each mesoNsZnO drug system (Table 2). Furthermore, these are very close to those employed in many scCO_2_-mediated processes reported in the literature, which employed the same drugs [9,15,25,26,27,28].

The contact time for the mesoNsZnO-CTZ system was selected according to a previous work [14], whereas that for the mesoNsZnO-IBU one, which resulted in the most promising one among those here reported, was varied from 3 h to 24 h to investigate its role on the final drug loading. The increment in contact time was preferred to that of temperature and pressure, since it has been reported that both an isothermal increase in pressure and an isobaric increase in temperature result in the lowering of the equilibrium drug loading [4].

In order to investigate the effect of the scCO_2_ on the mesoNsZnO structure, additional experiments were conducted on the sole carrier pellet, i.e., without the presence of the drug in the glass cylinder, both at the less severe (35 °C, 10 MPa, 8 h) and most severe (100 °C, 25 MPa, 12 h) working conditions adopted for the SSI process.

### 2.4. Characterization

Physical-chemical characterization of the samples was performed through X-ray diffraction (XRD), Fourier transform infrared spectroscopy (FTIR), field emission scanning electron microscopy (FESEM), and nitrogen adsorption analysis.

XRD patterns were obtained with a PANalytical X’Pert (Cu Kα radiation, Malvern Panalytical, Almelo, The Netherlands) diffractometer. Data were collected with a 2D solid state detector (PIXcel) from 10 to 80 2θ with a step size of 0.001 2θ and a wavelength of 1.54187 Å.

FTIR spectra were recorded on powders dispersed in potassium bromide with a Bruker Equinox 55 spectrometer (Bruker, Billerica, MA, USA) operating at 2 cm^−1^ resolution, after outgassing the sample at room temperature for 1 h (residual pressure equal to 0.1 Pa).

FESEM images were recorded with an FESEM Zeiss Merlin instrument equipped with an EDS detector (Oxford Instruments, Abingdon-on-Thames, UK).

Nitrogen adsorption isotherms were measured with a Quantachrome AUTOSORB-1 instrument (Quantachrome Instruments, Boynton Beach, FL, USA). Before the nitrogen adsorption, samples were outgassed at 40 °C for 2 h. Brunauer–Emmett–Teller (BET)-specific surface areas were calculated in the relative pressure range 0.04–0.1, and the pore size distribution was determined through the DFT (density functional theory) method, using the non-local density functional theory (NLDFT) equilibrium model for cylindrical pores [29].

### 2.5. Evaluation of the Drug Loading and the Number of Molecular Layers on the Carrier Surface

Ethanol extraction and ultraviolet (UV) spectroscopy were employed to quantify the amount of drug loading after SSI. 50 mg of each drug-loaded mesoNsZnO sample ($m_{tot}$) were contacted with 30 mL of ethanol under constant stirring (350 rpm) for 2 h at room temperature to extract the incorporated drug. After, the ZnO carrier was separated through three 30-min centrifugation cycles at 4000 rpm, and the alcoholic solution was subjected to UV spectroscopy to quantify the amount of drug loaded in the sample ($m_{drug}$). A Beckman–Coulter DU 730 Spectrophotometer was used (Beckman Coulter, Indianapolis, IN, USA). The drug loading was calculated according to the following equation:

(1) $$drug\;loading=\frac{m_{drug}}{m_{raw\;ZnO}},$$

where $m_{raw\;ZnO}=m_{tot}-m_{drug}$ is the amount of raw ZnO carrier in the sample.

Drug loading also allows the number of molecular layers (NML) of the drug on the carrier surface to be determined [4]:

(2) $$NML=drug\;loading\times \frac{N_{A}\times a_{drug}}{M_{drug}\times SSA_{raw\;ZnO}},$$

where $N_{A}$ is the Avogadro number, $SSA_{raw\;ZnO}$ is the specific surface area (SSA) of the carrier before drug incorporation, $M_{drug}$ is the drug molecular weight, and $a_{drug}$ is the projection area of the drug molecule. Table 3 reports different values of $a_{drug}$ available in the literature for IBU, CTZ, and HC.

## 3. Results

### 3.1. Characterization of the MesoNsZnO Carrier

**Figure 2:**
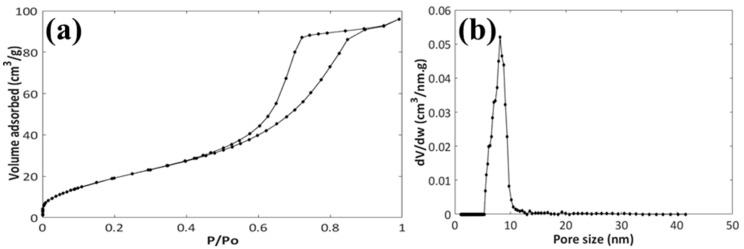
Nitrogen adsorption-desorption isotherms (**a**) and pore size distribution (**b**) of the mesoNsZnO carrier.

Figure 2 reports the nitrogen adsorption-desorption isotherms and the pore size distribution of the ZnO carrier. The isotherms (Figure 2a) are type IV according to IUPAC classification, which is associated with capillary condensation of nitrogen taking place in mesopores. An SSA of 75 m^2^/g and a pore volume of 0.14 m^3^/g were found. The pore size distribution was homogenous and centered at 8 nm, confirming the mesoporous nature of the ZnO material (Figure 2b).

**Figure 3:**
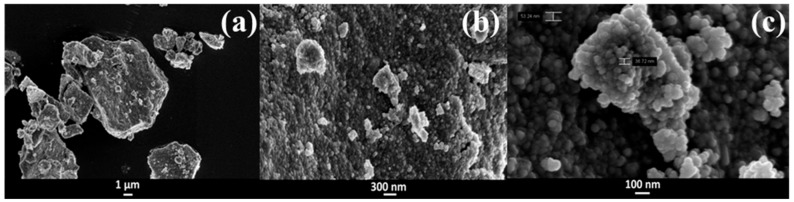
Field emission scanning electron microscopy (FESEM) images of the mesoNsZnO carrier at different magnification (**a**) 10 KX; (**b**) 50 KX; (**c**) 200 KX.

Figure 3 displays the FESEM images of the ZnO sample at low and high magnification. At the lower magnification, the material appears in the form of micrometric platforms (≈10 μm) without any precise shape. However, increasing the magnification, a sub-micrometric organization of these larger aggregates in smaller spherical nanoparticles with a size of 30–60 nm can be observed. The morphology obtained here can be considered suitable for a biological application, since it does not include any rod-like particles, which are often related to toxicological issues [16].

**Figure 4:**
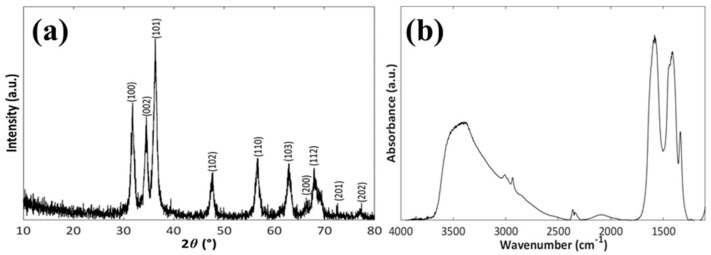
XRD pattern (**a**) and FTIR spectrum (**b**) of the mesoNsZnO carrier.

Figure 4 reports the XRD pattern and FTIR spectrum of the mesoNsZnO carrier. The XRD pattern (Figure 4a) reveals the highly crystalline single hexagonal phase of the wurtzite structure (JCPDS ICDD 36-1451). The characteristic peaks of the synthesis precursors do not appear in the XRD pattern, which means that pure ZnO can be obtained through the above-described synthetic method. The main peaks displayed in the FTIR spectrum (Figure 4b) are consistent with those reported in the literature [23]. In particular, the presence of O–H and C=O groups on the surface is evident. The broad band centered at about 3400 cm^−1^ is ascribed to the O–H stretching and the peaks in the 1700–1250 cm^−1^ range can be related to surface carbonate-like species.

### 3.2. Drug Loading and Characterization of the MesoNsZnO Drug Systems

Drug loading of the different mesoNsZnO drug systems, which was evaluated through ethanol extraction of the drug (Section 2.5), is reported in Table 4. IBU was found to exhibit the highest drug loading on the mesoNsZnO carrier, whereas no loading of HC could be detected. As far as CTZ is concerned, its drug loading is approximately half of IBU. In fact, a drug loading of 0.092 g/g was found for CTZ after 12 h of supercritical treatment and a value of 0.18 g/g was found for IBU after only 8 h.

**Figure 5:**
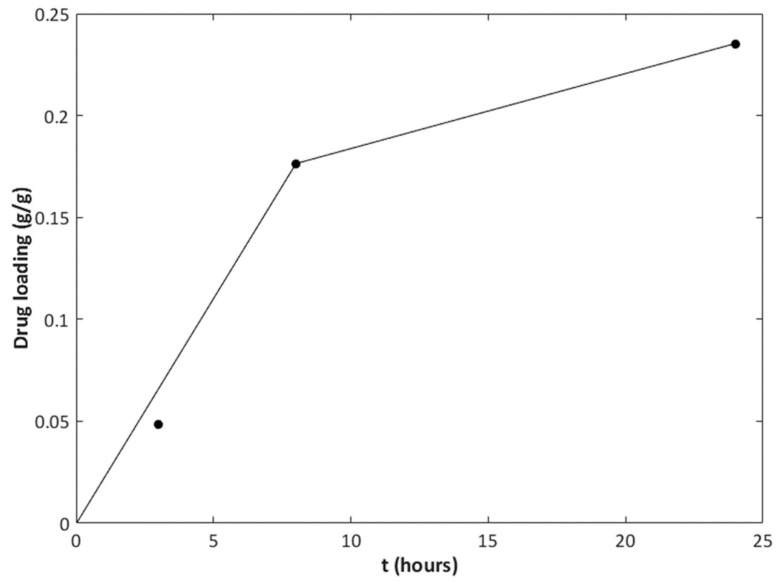
Drug loading of the mesoNsZnO-IBU system versus contact time.

Since IBU resulted in the highest drug uptake among the systems here investigated, SSI tests at different contact times were conducted, and the results are plotted in Figure 5. When the contact time was increased from 3 h to 8 h, the drug loading was more than triplicated; however, a further increase of the contact time from 8 h to 24 h only resulted in an additional 30% increment of the drug uptake. A maximum loading of 0.24 g/g was found for the mesoNsZnO-IBU system.

**Figure 6:**
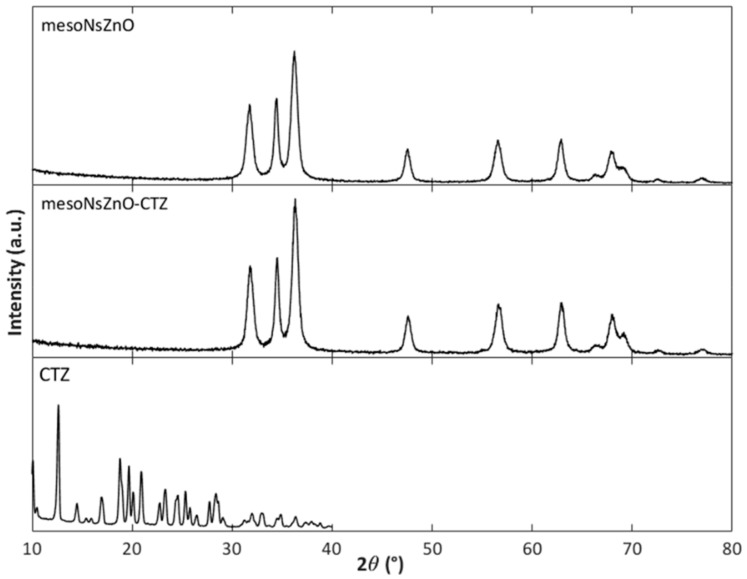
XRD patterns: MesoNsZnO carrier, mesoNsZnO-CTZ system, CTZ.

**Figure 7:**
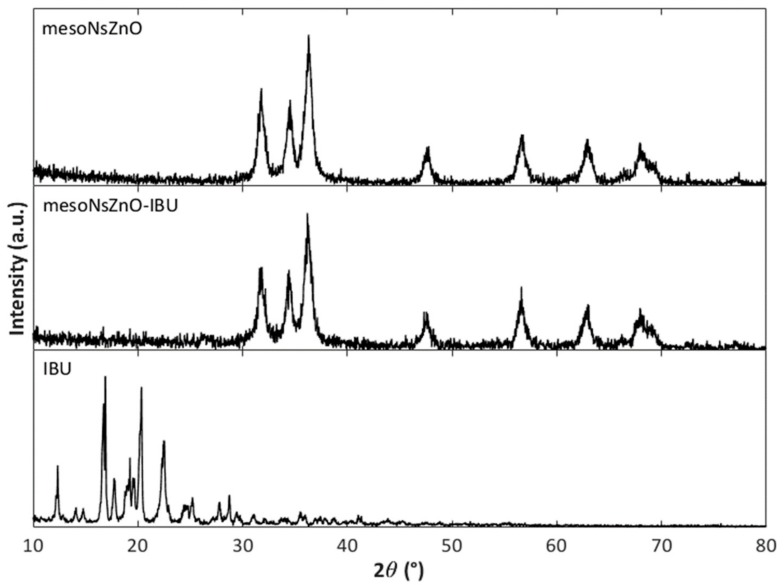
XRD patterns: MesoNsZnO carrier, mesoNsZnO-IBU system obtained after a contact time of 24 h, IBU.

XRD characterization of the drug-impregnated ZnO samples was conducted to investigate the physical state of the loaded drug. Figure 6; Figure 7 report the XRD patterns of the mesoNsZnO-CTZ and mesoNsZnO-IBU systems, which are compared with those of the mesoNsZnO carrier before impregnation and those of the pure drugs, respectively. In both cases, the drug-loaded materials show the typical hexagonal wurtzite structure, and no additional diffraction peaks of the crystalline drugs can be observed. This evidences that the loaded drug molecules are not assembled in the crystalline structure, and the interaction between the drug molecules and the ZnO surface results in the amorphization of the drug. The XRD patterns of the mesoNsZnO-HC system (not shown) did not also reveal any trace of drug crystals. In this case, this was not related to drug amorphization due to the negligible amount of this drug on the carrier, as reported in Table 4. The absence of drug crystals in this sample was ascribed to the very low solubility of HC in scCO_2_, which means that the amount of unadsorbed re-crystallized drug upon depressurization is probably below the detection limit of the instrument.

**Figure 8:**
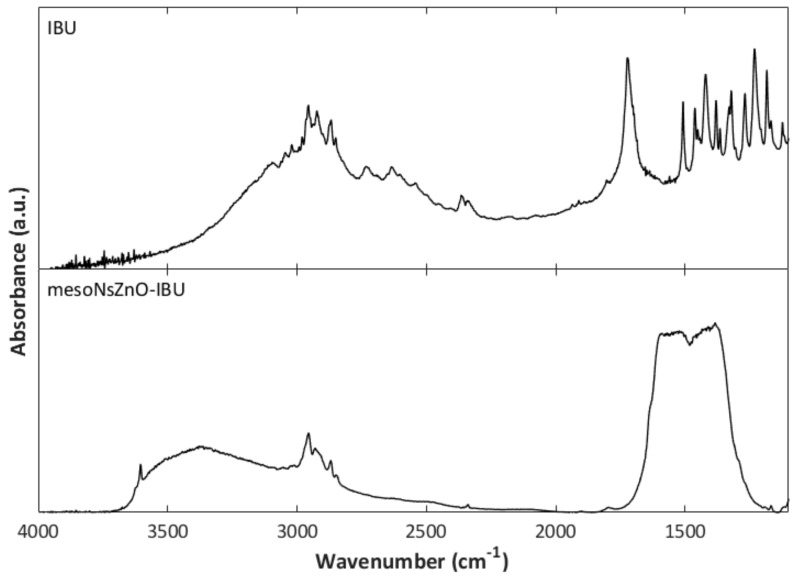
FTIR spectra of IBU and mesoNsZnO-IBU system obtained after a contact time of 24 h.

FTIR analysis can provide useful information about possible molecular interactions between the drug and ZnO surface, which may give reason for drug amorphization. This is particularly evident as far as the mesoNsZnO-IBU system is concerned. In Figure 8, the FTIR spectrum of the mesoNsZnO-IBU system obtained after a contact time of 24 h is compared with that of pure IBU. While the IBU peaks in the region of 2850–3000 cm^−1^, which is attributed to aliphatic C–H stretching vibrations of the drug molecule [30], are found also in the spectrum of the mesoNsZnO-IBU system, the same does not occur for the peak at 1721 cm^−1^, which is attributed to the stretching vibration of the carboxyl group [30]. The absence of this peak may be due to a depletion of carboxyl groups, due to the acid–base interaction with the ZnO surface, i.e., deprotonation and transformation into carboxylate species. Bands of carboxylate species are known to appear at lower frequencies [31] so that their intensity is masked by the peaks due to the surface carbonate-like species of ZnO in the 1700–1250 cm^−1^ range. This reveals a strong interaction between the carboxyl group of the drug and the ZnO surface. It has been reported [32], indeed, that co-processing of IBU and magnesium trisilicate resulted in absorbance reduction or even disappearance of the drug carboxyl group due to its possible acid–base interaction with the MgO of magnesium trisilicate. In analogy to the above-mentioned literature work, an acid–base interaction of the IBU carboxyl group and ZnO is suggested.

### 3.3. Effect of the scCO_2_ Treatment on the MesoNsZnO

The reaction between ZnO and CO_2_ to give ZnCO_3_ is a well-known phenomenon [33] and, for this reason, the effect of the stability of the mesoNsZnO carrier upon the supercritical treatment was investigated by exposing the sole carrier pellet to scCO_2_ both at the less severe (35 °C, 10 MPa, 8 h) and most severe (100 °C, 25 MPa, 12 h) working conditions adopted for the SSI process. The treated samples underwent XRD and FTIR analyses.

The collected XRD patterns (not shown) revealed that, in both cases, the hexagonal wurtzite pattern of ZnO was preserved, and no new peaks were detected, which points out that no extensive reaction between ZnO and the CO_2_ occurred.

**Figure 9:**
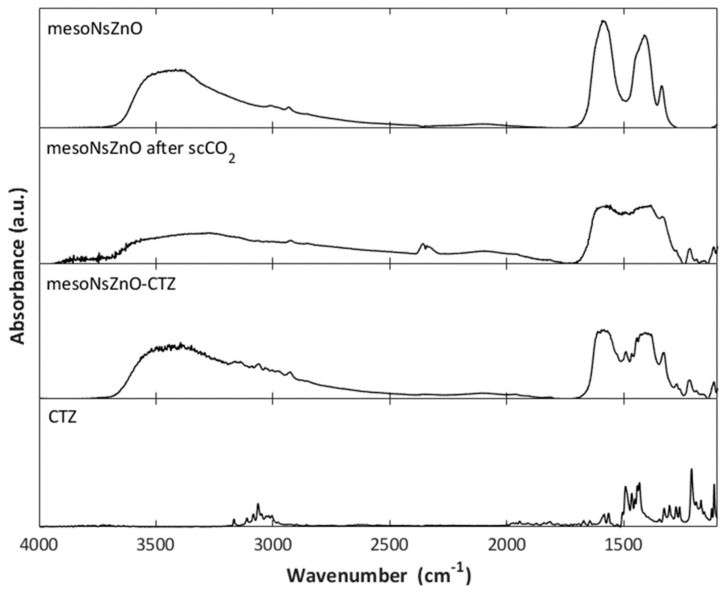
FTIR spectra: MesoNsZnO carrier, mesoNsZnO after the scCO_2_ treatment (100 °C, 25 MPa, 12 h), mesoNsZnO-CTZ system, CTZ.

The FTIR spectrum of the mesoNsZnO carrier after treatment at the less severe (35 °C, 10 MPa, 8 h) working conditions did not significantly differ from that of the untreated material reported in Figure 4b. On the other hand, some changes in the FTIR spectrum were found when the material was processed at the most severe working conditions (100 °C, 25 MPa, 12 h). Figure 9 compares the FTIR spectrum of mesoNsZnO after the scCO_2_ treatment and that of the mesoNsZnO-CTZ system with those of the unprocessed carrier and the pure drug. The FTIR spectrum of the sole ZnO carrier after the supercritical treatment shows an intensity increase of the absorption in the 1600–1000 cm^−1^ range due to surface carbonate-like species caused by the adsorption of CO_2_ on the ZnO surface [34]. However, the spectrum of the mesoNsZnO-CTZ system, which underwent the same supercritical treatment, shows a significantly less intense adsorption in the same region. It can be concluded that during SSI the presence of the adsorbed drug played a protective role versus the ZnO surface so reducing the chemisorption of CO_2_.

## 4. Discussion

The obtained results have pointed out that SSI of the mesoNsZnO carrier with IBU and CTZ in their amorphous form is feasible and significant drug loading can be obtained. On the other hand, HC loading could not be achieved. Even though this last result is negative, it could yield more insight into the features affecting drug adsorption from supercritical solutions, since studies with null effect are rarely reported in the literature [4].

HC is a poorly scCO_2_-soluble drug (solubility expressed as mole fraction ≤10^−6^) and its consequent low concentration in the supercritical solvent may contribute to limit its uptake in the adsorbent material [4]. However, this should not necessarily imply a negligible loading of this drug [4]. An explanation of this result can be attempted by considering both the drug-drug and the drug-ZnO molecular interactions during the supercritical treatment. Table 1 reports the *n*-octanol water partition coefficient (log*P*) and the number of hydrogen bond donors and acceptors (HBDA) of the three drugs, which can be considered a measure of the hydrophobicity and hydrophilicity of a substance. The table shows that HC displays the lowest log*P* and highest HBDA values, respectively, which points out that this drug is the most hydrophilic one among those here investigated. This gives reason for the scarce solubility of HC in the non-polar scCO_2_ but may also explain the unsuccessful loading of the mesoNsZnO carrier. In fact, the existence of strong drug-drug molecular interactions during SSI probably prevents those between the drug and the carrier to occur. To support this hypothesis, some impregnation tests of the ZnO carrier with HC were conducted in the presence of ethanol, which is a solvent where HC displays a high solubility. The XRD patterns of the impregnated sample (not shown) revealed a significant presence of drug crystals, which suggests that the drug-ZnO molecular interactions were not strong enough to achieve the amorphization of the drug.

Table 4 reports that the IBU loading on the mesoNsZnO carrier is approximately double than that of CTZ. This may occur because IBU has higher solubility (≥1 × 10^−3^) than CTZ (10^−5^–10^−3^) in scCO_2_, which results in higher drug concentration in the supercritical solvent during SSI. Furthermore, since the hydrophobicity of these two drugs is comparable and much higher than that of HC, the drug loading difference could be related to their molecular size. Table 1 reports the van der Waals volume of the drugs, which gives an idea of the hindrance of each molecule. Being smaller than CTZ, IBU could more easily penetrate the mesoNsZnO porous structure, and this could probably give reason for the higher loading of this drug.

The calculation of the number of molecular layers (NML) of the drug on the carrier surface is reported in Table 5 for the successfully impregnated systems. This calculation is strongly affected by the $a_{drug}$ value, for which different sources are available in the literature. However, data in Table 5 suggest that, regardless of the adopted $a_{drug}$ value, the NML generally exceeds unity and increases with contact time (mesoNsZnO-IBU system). According to the literature [4] both adsorption and precipitation contribute to the overall drug loading of non-swellable matrices, and when the NML exceeds unity this may be an indication of the drug precipitation within the pores. However, it must be stressed that even though drug precipitation occurs giving rise to up to three to eight molecular layers on the carrier surface, the drug is still confined in its amorphous form (Figure 6 and Figure 7).

Previous works reviewed by Gurikov and Smirnova [4] for IBU-mesoporous-silica or IBU-silica-aerogel systems report that crystalline drug is always detected when NML exceeds unity, the only exception being an amorphous-IBU-hydrophilic-silica system for which an NML = 3.8 was calculated [35]. On the other hand, it is well-accepted that porous media have a size-constraint effect on nucleation and crystal growth of hosted species [36] because the spatial constraints of a capillary are imposed on the clusters of molecules before they reach the critical nucleation size, so that nucleation and growth will be prevented, and the system will exist in an intrinsically non-crystalline state. This vitrification mechanism was firstly reported by Jackson and McKenna [37].

The results obtained here point out that even though mesoNsZnO exhibits lower specific surface area than silica carriers, due to its porous structure, it is capable to confine an amount of drug corresponding to a high number of drug molecular layers preserving the amorphous character of the adsorbed drug. This may be exploited to prepare pharmaceutical formulations where the solubility and dissolution rate of poorly water-soluble drugs is significantly increased.

## 5. Conclusions

A mesoporous ZnO carrier with uniform pores of 8 nm size, a spherical-nanoparticle morphology, and an SSA of 75 m^2^/g has been subjected to SSI to achieve incorporation of poorly water-soluble drugs for the first time. Successful impregnation results were obtained when IBU and CTZ were employed, whereas negligible drug loading was obtained with HC. The different solubility in the supercritical medium, different size, and polarity of the drug may give reason for the different behavior of the three systems. In particular, the drug-drug molecular interactions should not be too strong to prevent those between the drug and the porous matrix to occur during the impregnation process. Instead, if these interactions occur, confinement of amorphous drug may result in an amount that corresponds to multilayer coverage. The supercritical treatment did not affect the structure of the mesoNsZnO, and the presence of the adsorbed drug exerted a protective role since it reduced the chemisorption of CO_2_ on the surface of the carrier. The mesoNsZnO-IBU system resulted in the highest drug loading, which is approximately double than that of the mesoNsZnO-CTZ one. An investigation of the role of the contact time on the drug loading of the mesoNsZnO-IBU system showed that while the drug loading was triplicated between 3 and 8 h an additional 30% increment was achieved between 8 and 24 h. A maximum loading of 0.24 g/g was found for the mesoNsZnO-IBU system, which corresponds to 3–8 molecular layers on the carrier surface.

The obtained results evidence that mesoporous ZnO could be exploited to prepare topical pharmaceutical formulations by means of SSI of poorly water-soluble drugs. Future work should be focused on in vitro drug release and over-time stability investigation of the drug formulations.

## Figures and Tables

**Table 1:** Chemical structure, *n*-octanol water partition coefficient (log*P*), number of hydrogen bond donors and acceptors (HBDA), and van der Waals volume.

Drug	Chemical Structure	log*P* ^1^	HBDA ^2^	van der Waals Volume(Å^3^) ^2^
Ibuprofen (IBU)	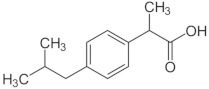	3.97	3	211.80
Clotrimazole (CTZ)	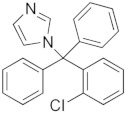	6.1	1	306.59
Hydrocortisone (HC)	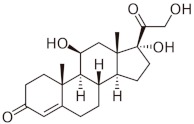	1.61	8	347.26

^1^ Taken from PubChem [21]; ^2^ Calculated with free online service chemicalize.com [22].

**Table 2:** Working conditions of the SSI for the different mesoNsZnO drug systems.

Drug	T (°C)	P (MPa)	Contact Time (h)
Clotrimazole (CTZ)	100	25	12
Hydrocortisone (HC)	45	13	8
Ibuprofen (IBU)	35	10	3, 8, 24

**Table 3:** Different values of $a_{drug}$ available in the literature for IBU, CTZ, and HC.

Drug	$a_{drug}\;({Å}^{2}){\;}^{1}$	$a_{drug}\;({Å}^{2}){\;}^{2}$	$a_{drug}\;({Å}^{2}){\;}^{3}$
Ibuprofen (IBU)	89.20	35.44	64.57
Clotrimazole (CTZ)	117.61	66.11	75.55
Hydrocortisone (HC)	-	47.67	97.02

^1^ Calculated from effective molecule diameter, which is estimated from the solvent accessible surface area, taken from [4]. ^2^ Minimum of the projection areas of the conformer, based on the van der Waals radius, calculated with free online service chemicalize.com [22]. ^3^ Maximum of the projection areas of the conformer, based on the van der Waals radius, calculated with free online service chemicalize.com [22].

**Table 4:** Drug loading of the different mesoNsZnO drug systems.

Drug	T (°C)	P (MPa)	Contact Time (h)	Drug Loading (g/g)
Clotrimazole (CTZ)	100	25	12	0.092
Hydrocortisone (HC)	45	13	8	nil
Ibuprofen (IBU)	35	10	3	0.048
			8	0.18
			24	0.24

**Table 5:** NML of the drug on the carrier surface calculated with Equation (2) with different values of $a_{drug}$.

Drug	Contact Time (h)	*NML* ^1^	*NML* ^2^	*NML* ^3^
Clotrimazole (CTZ)	12	2.5	1.4	1.6
Ibuprofen (IBU)	3	1.7	0.66	1.2
	8	6.1	2.4	4.4
	24	8.2	3.2	5.9

^1^$a_{drug}$ (Table 3) is calculated from effective molecule diameter, which is estimated from the solvent accessible surface area, taken from [4]. ^2^ $a_{drug}$ (Table 3) is the minimum of the projection areas of the conformer, based on the van der Waals radius—calculated with free online service chemicalize.com [22]. ^3^ $a_{drug}$ (Table 3) is the maximum of the projection areas of the conformer, based on the van der Waals radius, calculated with free online service chemicalize.com [22].
